# Human Pupillary Dilation Response to Deviant Auditory Stimuli: Effects of Stimulus Properties and Voluntary Attention

**DOI:** 10.3389/fnins.2016.00043

**Published:** 2016-02-17

**Authors:** Hsin-I Liao, Makoto Yoneya, Shunsuke Kidani, Makio Kashino, Shigeto Furukawa

**Affiliations:** ^1^Human Information Science Laboratory, NTT Communication Science Laboratories, NTT CorporationAtsugi, Japan; ^2^Department of Information Processing, Interdisciplinary Graduate School of Science and Engineering, Tokyo Institute of TechnologyYokohama, Japan

**Keywords:** audition, oddballs, novelty, attention, locus coeruleus, norepinephrine

## Abstract

A unique sound that deviates from a repetitive background sound induces signature neural responses, such as mismatch negativity and novelty P3 response in electro-encephalography studies. Here we show that a deviant auditory stimulus induces a human pupillary dilation response (PDR) that is sensitive to the stimulus properties and irrespective whether attention is directed to the sounds or not. In an auditory oddball sequence, we used white noise and 2000-Hz tones as oddballs against repeated 1000-Hz tones. Participants' pupillary responses were recorded while they listened to the auditory oddball sequence. In Experiment 1, they were not involved in any task. Results show that pupils dilated to the noise oddballs for approximately 4 s, but no such PDR was found for the 2000-Hz tone oddballs. In Experiments 2, two types of visual oddballs were presented synchronously with the auditory oddballs. Participants discriminated the auditory or visual oddballs while trying to ignore stimuli from the other modality. The purpose of this manipulation was to direct attention to or away from the auditory sequence. In Experiment 3, the visual oddballs and the auditory oddballs were always presented asynchronously to prevent residuals of attention on to-be-ignored oddballs due to the concurrence with the attended oddballs. Results show that pupils dilated to both the noise and 2000-Hz tone oddballs in all conditions. Most importantly, PDRs to noise were larger than those to the 2000-Hz tone oddballs regardless of the attention condition in both experiments. The overall results suggest that the stimulus-dependent factor of the PDR appears to be independent of attention.

## Introduction

Pupillary responses under constant illumination are known to reflect not only emotional arousal (Partala and Surakka, 2003; Bradley et al., 2008) but also cognitive functions such as attention (Privitera et al., 2010; Gabay et al., 2011; Binda et al., 2013; Eldar et al., 2013), memory (Goldinger and Papesh, 2012; Naber et al., 2013), processing load (Kahneman and Beatty, 1967; Beatty, 1982; Koelewijn et al., 2015), preference (Yoshimoto et al., 2014), and decision making (Einhäuser et al., 2008, 2010; Preuschoff et al., 2011; Lavin et al., 2014). The cognitive functions are presumably modulated by the activation of the locus coeruleus–norepinephrine (LC-NE) system (Aston-Jones and Cohen, 2005; Sara, 2009). One major function of norepinephrine is to modulate the “fight-or-flight” response of the organism. In order for it to do so, the sympathetic nervous system needs to monitor any change in the environment, i.e., it must be sensitive to novel signals (e.g., Dayan and Yu, 2006). It is thus hypothesized that pupillary responses, reflecting the norepinephrine modulation, are sensitive to novel signals.

Indeed, a unique sound that deviates from a repetitive background sound is known to induce signature neural responses, such as mismatch negativity (MMN, Näätänen et al., 1978, 2007) and P300 (Squires et al., 1975; Donchin, 1981) in human electro-encephalography (EEG) recordings, and the unadapted neural responses in contrast to the stimulus-specific adaptation (SSA) to the repetitive sounds at the cellular level in mammals (Javitt et al., 1994; Ulanovsky et al., 2003; Patel et al., 2012). The mechanisms thought to underlie the deviant sound effect (i.e., the oddball effect) include adaptation to the repetitive sounds (Jääskeläinen et al., 2004; Ayala and Malmierca, 2012), mismatch between the prediction based on the memory trace and the on-line sensory input (Näätänen, 1992; Näätänen and Winkler, 1999), and the integrated framework of intra-areal adaptation and inter-areal lateral connections (Garrido et al., 2009). Moreover, the evidence obtained from sophisticated analysis of the MMN and P300 components (e.g., Escera et al., 1998; Polich, 2007) suggests that the acoustic novelty and change are detected through different underlying mechanisms such as the transient-detector mechanism that is related to preattentive-perceptual processing and revealed in N1 component, the change-detector mechanism that is related to stimulus-driven attention orienting and revealed in MMN and/or novelty P3a responses, and the attention mechanism that is related to subsequent memory processing in P3b response.

Previous pupillometry research has shown that pupillary responses are induced by the presentation of auditory stimuli, with various manipulations of the stimulus probability (Friedman et al., 1973; Qiyuan et al., 1985), property (Maher and Furedy, 1979; Steiner and Barry, 2011), and intensity (Stelmack and Siddle, 1982); For example, Steiner and Barry (2011) presented tones with a stimulus onset asynchrony of several seconds. After 10 repetitions of the same tone, another tone with a different frequency was presented. Results showed that the pupillary responses habituated for the repeatedly presented tones and recovered when the novel tone was presented (also see Maher and Furedy, 1979; cf. Stelmack and Siddle, 1982). The results suggest that pupillary responses reflect the change in the stimulus property.

However, it remains unclear how the pupil responds to acoustic novelty and change. In other words, how does pupil respond when there are different types of deviant sounds presented? It is an important question since it solves an issue whether the sound-induced pupillary response reflects a mechanism that detects any transient change *per se*, regardless of the content of the change, or whether the novelty and/or stimulus salience matters. Relatedly, does attention modulate the sound-induced pupillary response? Since the pupillary response is known to reflect the LC-NE modulation (Aston-Jones and Cohen, 2005), the investigation would also provide us insight of the neurotransmitter actions associating with the event-related potentials (ERPs) that is related to acoustic novelty and change detection.

In the current study, we examined whether human pupillary responses are induced by novel auditory stimuli, and if they are, whether and how the stimulus property (related to stimulus salience) and voluntary attention play a role there. In an auditory oddball sequence, we presented white noises and 2000-Hz tones as oddballs against repeated 1000-Hz tones. In three experiments, participants' pupillary responses were recorded while they listened to the auditory sequence. In Experiment 1, they were not involved in any task, and we examined whether the deviant oddballs induce a pupillary response and whether the stimulus properties of the oddballs matter. In Experiments 2 and 3, participants performed a discrimination task on the auditory oddballs or on visual oddballs while trying to ignore the stimuli from the other modality. The visual oddballs were Gabor patches or random-dot noise disks, presented uncorrelated with the auditory oddballs in random order. We examined whether attention plays a role in the pupillary response to auditory oddballs and, if so, how.

## Materials and methods

### Participants

Thirty-six people (aged from 21 to 43; median of 36 years old, 17 males) with normal or corrected-to-normal vision and normal hearing acuity were paid to participate in the current study (ten in Experiment 1; eight in Experiment 2; 18 in Experiment 3). All participants were naïve about the purpose of the current study. All the procedures were approved by the NTT Communication Science Laboratories Ethical Committee, and all participants gave informed written consent before the experiments.

### Stimuli and apparatus

Auditory stimuli were generated by a personal computer (Dell OptiPlex 755), transformed by an audio interface (Roland OCTA-CAPTURE), amplified with a headphone amplifier (Grace Design m903), and presented through a headphone (Sennheiser HD 595). Three types of auditory stimuli were used: a 1000-Hz tone, a 2000-Hz tone, and a white noise burst, all with the duration of 50 ms (including 5-ms raised cosine ramps) and the sampling rate of 44,100 Hz. All auditory stimuli had A-weighted sound pressure levels of 65 dB. The sound pressure levels were measured by a measuring instrumentation amplifier (Brüel and Kjær, 2636) that received input from the headphone.

**Figure 1:**
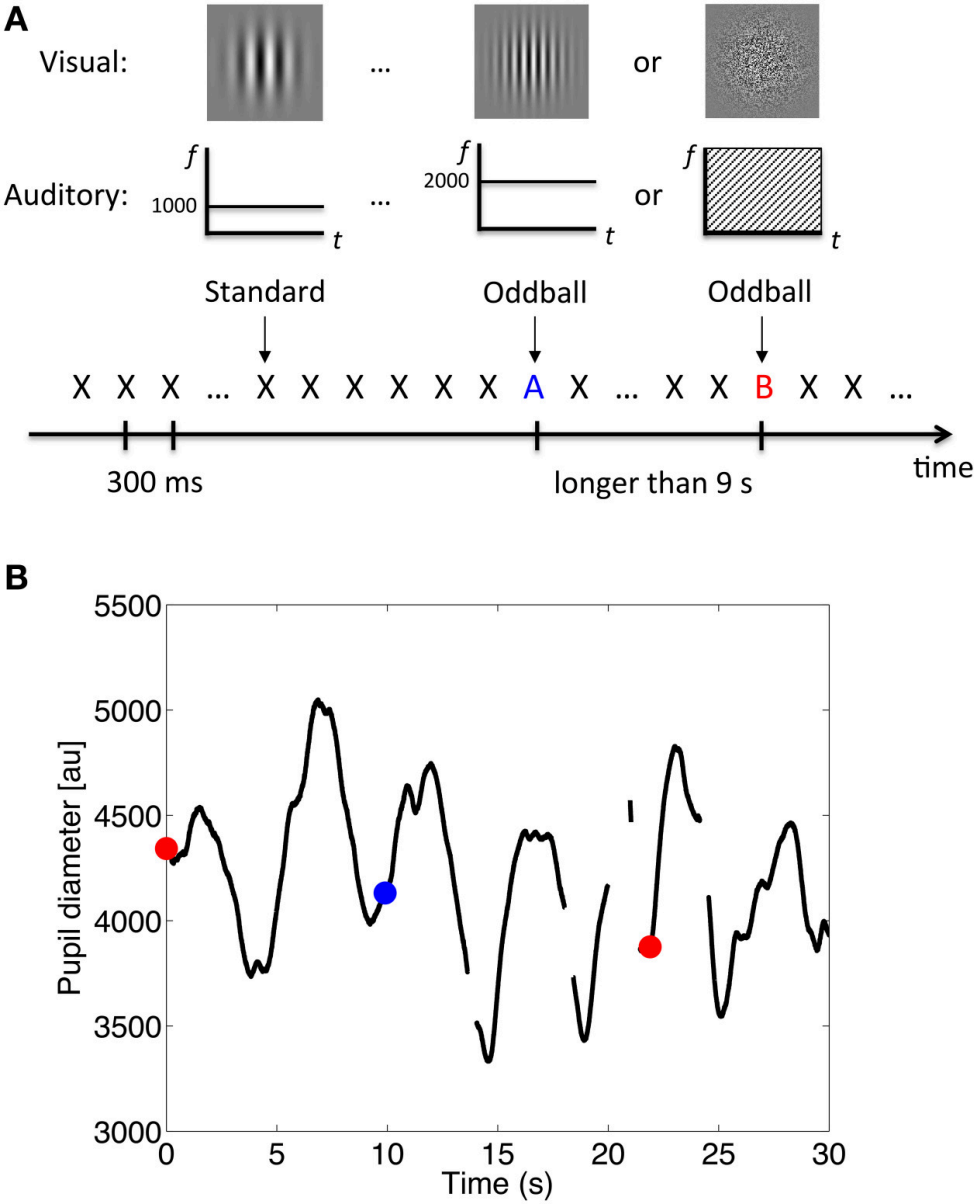
**Procedure and example of the results. (A)** Schematic illustration of the stimulus sequence, where X, A, and B, represent the auditory and visual stimuli. **(B)** Example of pupil diameter changes over time. Data during blinks were treated as missing (the off-line segments). The red dots represent the onset of the white-noise oddballs; the blue dot represents the onset of the 2000-Hz-tone oddballs.

Visual stimuli were generated by the same personal computer and presented on an 18.1-inch monitor (EIZO FlexScanL685Ex) with a frame rate of 60 Hz and resolution of 1280 × 1024 pixels. All visual stimuli were presented at the center of the monitor against a light gray background (12.9 cd/m^2^ in Experiment 1; 27.0 cd/m^2^ in Experiments 2 and 3). Four types of visual stimuli were used: a fixation point, coarse-grating Gabor patch, fine-grating Gabor patch, and random-dot noise disk. The fixation point was a small dark gray dot (0.25 × 0.25°, 0.33 cd/m^2^). The other visual stimuli were 5 × 5° in size with the mean luminance matched to the light gray background. The Gabor patches were generated by superimposing a Gaussian and a sine-wave function with a vertical orientation. The frequencies of the coarse-grating Gabor patch and the fine-grating Gabor patch were 1 cycle and 2 cycles per degree, respectively. The random-dot noise disk was generated by superimposing a Gaussian function and a random-dot pattern similar to that used by Julesz (1971). Each pixel had a 50% probability of being white or black (see Figure 1A for an illustration).

Behavioral responses were collected from a response box controlled by the same personal computer with a real-time mobile processor (Tucker-Davis Technologies, Inc. System III RM1). The response box had four buttons on it, aligned horizontally. All stimuli presentations and response recordings were controlled by MATLAB (The MathWorks, Inc.).

### Design

In an auditory sequence, oddballs were presented against repeated standard sounds. Oddball type (tone, noise) was manipulated as a within-subjects factor. The standard sound was the 1000-Hz pure tone, and the oddball was the 2000-Hz pure tone or the white noise. The inter-stimulus-interval was 300 ms. Each type of oddball was presented 40 times, for 80 oddball trials in total. There were 40 standard sounds coded as dummy oddballs and presented with the 80 real oddballs in an interval jittered in 9–12 s within the same auditory sequence in randomly assigned order. As a result, each oddball was separated by an interval longer than 9 s to avoid the effect of accumulated pupillary responses, and the distance between the real oddballs could be quite discrete over several tens of seconds (when one or more the dummy-coded standard tone oddballs were presented in-between). Since the interval was jittered, the total length of the auditory sequence was adjusted, and as a result the total number of standard sounds varied trial-by-trial. The mean number of standard sounds was 4250 in Experiment 1 (range from 4211 to 4284). The total duration of the auditory sequence was around 20 min.

In Experiment 1, the fixation point was presented at the center of the monitor (i.e., the fixation display) throughout the experiment. In Experiment 2, a visual sequence was presented simultaneously with the auditory sequence. The Gabor patches and the random-dot noise disk were presented for 50 ms and synchronized with the auditory stimuli. The screen in-between the Gabor patches and the random-dot noise disk was the fixation display. The coarse-grating Gabor patch was always presented simultaneously with the standard 1000-Hz pure tone. The fine-grating Gabor patch and the random-dot noise disk were used as visual oddballs and presented simultaneously with the auditory oddballs. However, the types of visual-oddball (the fine-grating Gabor patch or the random-dot noise disk) and auditory-oddball (the 2000-Hz tone or the white noise) stimuli were unrelated; namely, the combination of the visual and auditory oddballs was randomized for each trial. Unlike Experiment 1, there was no dummy-coded standard sounds oddball mixed with the real oddballs for the oddball presentation. In this case, all the 80 real oddballs, which consisted of the two types of oddballs, were presented in an actual interval jittered in 9–12 s in randomly assigned order. The mean number of standard sounds was 2774 (range from 2752 to 2807).

In Experiment 3, the auditory and visual sequences were presented as in Experiment 2 except that the visual and auditory oddballs were always presented at different times. The visual oddballs were presented at various latencies within a 4800-ms time window centered at the latency of the auditory oddballs. The mean number of standard sounds was 2769 (range from 2736 to 2825).

In Experiments 2 and 3, the attention condition (attend audition or attend vision) was manipulated as a within-subject factor in separate blocks in counterbalanced order. In the attend-audition condition, participants were asked to discriminate whether the auditory oddball was the 2000-Hz pure tone or white noise and ignore the visual stimuli. In the attend-vision condition, they were asked to discriminate whether the visual oddball was the fine-grating Gabor patch or random-dots noise disk and ignore the auditory stimuli.

### Procedure

All participants were given written and oral explanations of the nature of the experiment and the pupillary response recording. Participants sat in front of the monitor at a viewing distance of 80 cm in a dimly lit chamber, with their head fixed on a chinrest. Their pupil responses were recorded while the auditory oddball sequence was presented diotically through the headphone.

In Experiment 1, participants were asked to fixate the central fixation point throughout the experiment. They were not involved in any task; they just listened to the auditory sequence. In Experiments 2 and 3, they were asked to perform the discrimination task of the oddballs as soon and as accurately as possible by pressing corresponding buttons on the response box.

In Experiment 2, the stimulus-response match was fixed. In the attend-audition condition, all the participants were asked to press the right-most button in the response box with their right hand when they heard the noise oddball and press the left-most button in the response box with their left hand for the 2000-Hz tone oddball. In the attend-vision condition, they were asked to press the right-most and left-most buttons for the random-dots noise disk and fine-grating Gabor patch visual oddballs, respectively.

In Experiment 3, the stimulus-response match was counterbalanced across participants. Half participants used their right hand to press the right-most button for the 2000-Hz tone oddball in the attend-audition condition and for the fine-grating Gabor patch in the attend-vision condition. Accordingly, they used their left hand to press the left-most button for the noise oddballs in the attend-audition condition and the random-dot noise disk in the attend-vision condition.

### Pupil size measurement

Pupil diameter was measured binocularly with an infrared eye-tracker camera (Eyelink 1000 Desktop Mount, SR Research Ltd.) with a sampling rate of 1000 Hz. The camera was positioned just below the monitor. The standard five-point calibration procedure for the Eyelink system was conducted prior to each auditory sequence block. After the calibration, there was a 30-s waiting period before the start of the auditory sequence presentation. Participants were asked to fixate the central fixation point to adapt to the constant luminance. They were instructed to blink naturally during the experiment.

In each condition, certain standard-sound trials—40 in each condition—were chosen to serve as the baseline of the pupillary response to repeatedly presented auditory stimuli. In Experiment 1, the 40 standard sounds that were coded as the dummy oddballs were chosen. In this case, the intervals between the chosen standard-sound trials and oddballs were controlled to be longer than 9 s (jittered in 9–12 s) to avoid the effect of accumulated pupillary responses across trials within the analysis window (-1 to 4 s to stimulus onset). In Experiments 2 and 3, the chosen standard-sound trials were those that appeared 4.5 s after the oddball sounds because we found that the sound-evoked pupillary response in Experiment 1 decayed after 4 s of stimulus onset. Since the limit of the oddball interval was 9 s, the chosen standard-sound trials were not involved in the effect of accumulated or residual pupillary responses induced by the oddballs that appeared before or after them, or vice versa.

## Results

Data acquired during blinks were treated as missing. Figure 1B shows an illustration of pupil diameter changes over time. Only right-eye data were analyzed, since data from both eyes showed a similar pattern. To reduce signal jitter due to the over-fine sampling rate for pupil diameter measurement, we reduced the sampling rate for analysis to 10 Hz. We kept the data points for every 100 ms while dropping the data in-between without any filtering process.

The Eyelink 1000 system output arbitrary units [au] in the range of 400 to16,000 units to represent pupil diameters, which are known to normally be in the range of 3–9 mm with individual differences. The mean arbitrary unit across participants in all experiments was 6300 with a standard deviation of 1629. The arbitrary units were not calibrated and were susceptible to influence from the tracking setup. To compare the results across conditions, for each trial, we conducted baseline correction by subtracting the mean of the data during the 1-s period before the stimulus onset from the raw data.

Pupil diameter data for the reference period (i.e., 1-s period before the stimulus onset) had a symmetrical long-tailed distribution over participants and did not fit the normal distribution (Kolmogorov-Smirnov goodness-of-fit test, *p*s < 0.001). To avoid undesirable contributions of outliers, we calculated the median, instead of the mean, to represent the pupil diameter change across time.

### Pupil diameter change time locked to stimulus onset

**Figure 2:**
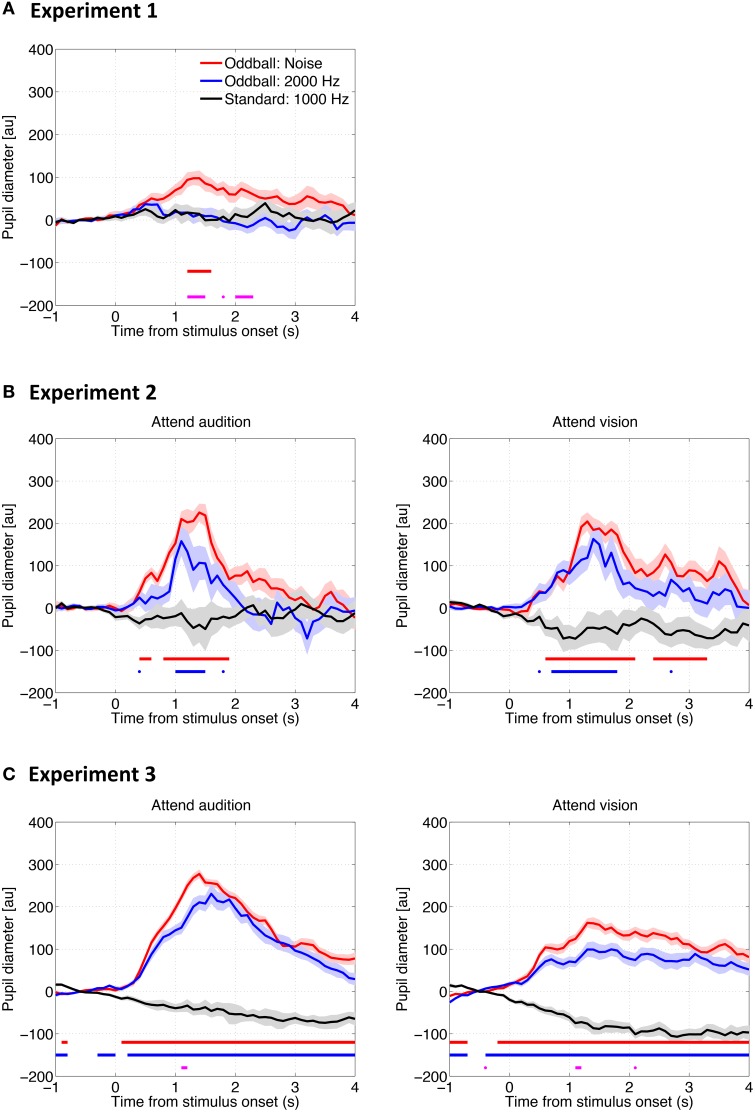
**Pupil diameter change as a function of time relative to the auditory stimulus onset in Experiment 1 (A), Experiment 2 (B), and Experiment 3 (C)**. The solid lines represent the median of the pupil diameter derived from all trials for all participants as a function of time relative to the auditory stimulus onset. The shadows represent the standard error derived from a bootstrapping procedure (resampling *n* = 1500). The horizontal red and blue lines represent statistical differences between the baseline (i.e., the response to the standard tones) and the noise and 2000-Hz oddballs, respectively. The horizontal magenta line represents the difference between the two types of oddballs (bootstrapping, with the Bonferroni correction).

Figure 2 shows the pupil diameter change as a function of time relative to the auditory stimulus onset in all three experiments. To examine whether pupil diameter reliably increased, i.e., the pupillary dilation response (PDR), we conducted a bootstrapping procedure (resampling *n* = 1500) at each time point to mark the time period in which the pupil diameter change was significantly larger than the baseline, i.e., the pupillary response to the standard tones (criterion: *p* = 0.0006, adjusted by the Bonferroni correction). Results showed that the PDR to the noise oddball was significantly larger than the baseline and the dilation remained for several seconds in all experiments, regardless of whether a task was required or not or regardless of the attention condition. In contrast, the PDR to the 2000-Hz tone oddball was only observed when a task was required (Experiments 2 and 3), but was not found in the passive listening condition (Experiment 1; no horizontal blue line/point in Figure 2A).

### PDR: stimulus property and voluntary attention

**Figure 3:**
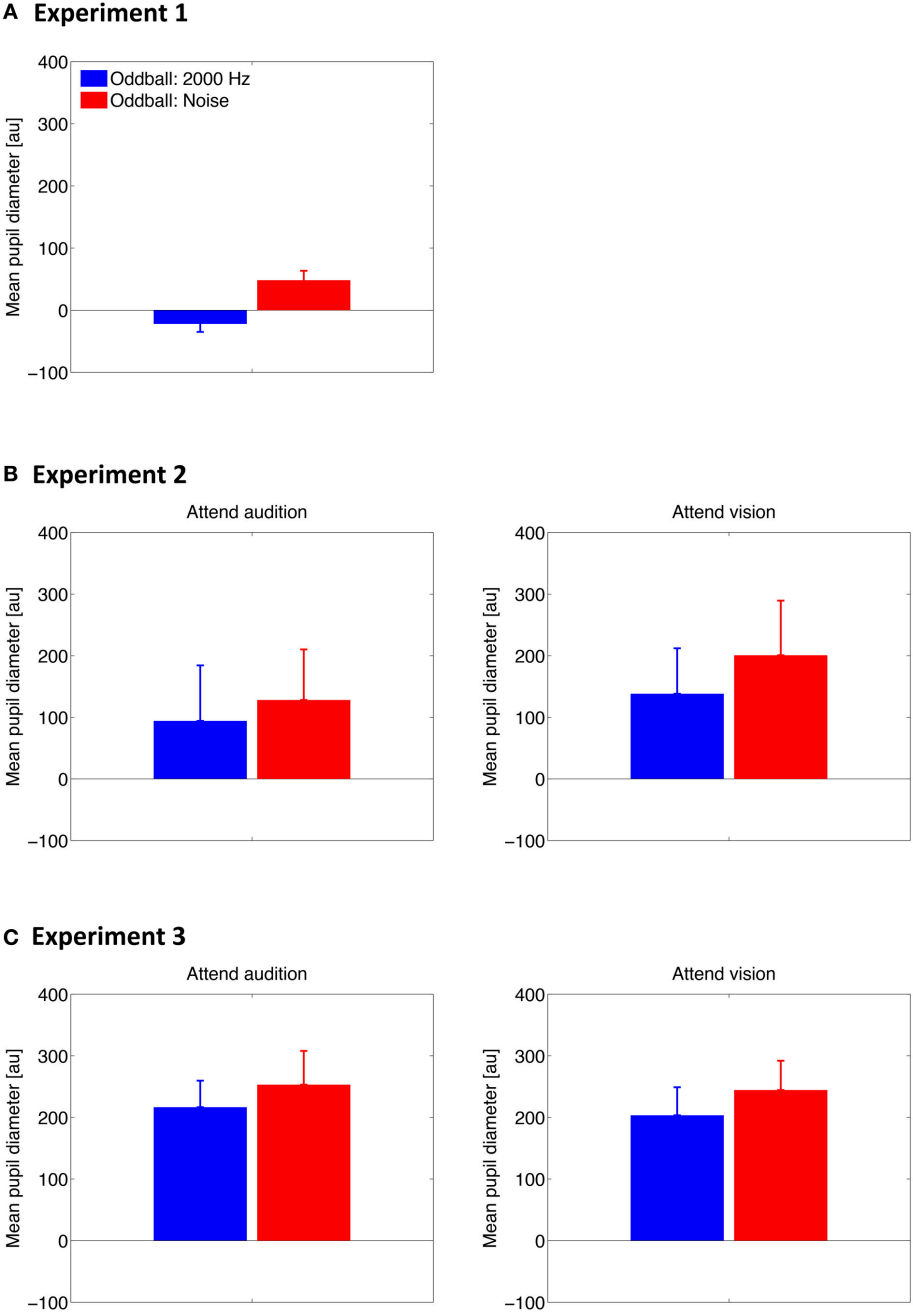
**Mean of the pupil diameter over 0–4 s after the stimulus onset across participants in Experiment 1 (A), Experiment 2 (B), and Experiment 3 (C)**. Error bars represent standard errors across participants.

To examine whether the PDR to auditory oddballs differs between the oddball type and whether voluntary attention plays a role, we averaged the pupil diameter for the two types of oddballs along 0–4 s to represent the mean PDR for individual participants. Results are shown in Figure 3. The mean PDR was subjected to a paired Student's *t*-test in Experiment 1 and to a two-way repeated-measures analysis of variance (ANOVA) with oddball type (2000-Hz, noise) and attention condition (attend audition, attend vision) as within-subjects factors in Experiments 2 and 3.

Results showed that the mean PDR was larger for the noise oddballs than for the tone oddballs in all experiments [*t*_(9)_ = 4.30, *p* = 0.002 in Experiment 1; *F*_(1, 7)_ = 10.88, *p* = 0.013 in Experiment 2; *F*_(1, 17)_ = 19.94, *p* < 0.001 in Experiment 3]. The effect of oddball type did not interact with the attention condition in Experiment 2 [*F*_(1, 7)_ = 0.26, *p* = 0.63] or Experiment 3 [*F*_(1, 17)_ = 0.03, *p* = 0.87]. Furthermore, the mean PDR did not differ between the attention conditions [*F*_(1, 7)_ = 2.66, *p* = 0.15 in Experiment 2; *F*_(1, 17)_ = 0.05, *p* = 0.83 in Experiment 3].

### Pupillary response to visual stimuli

**Figure 4:**
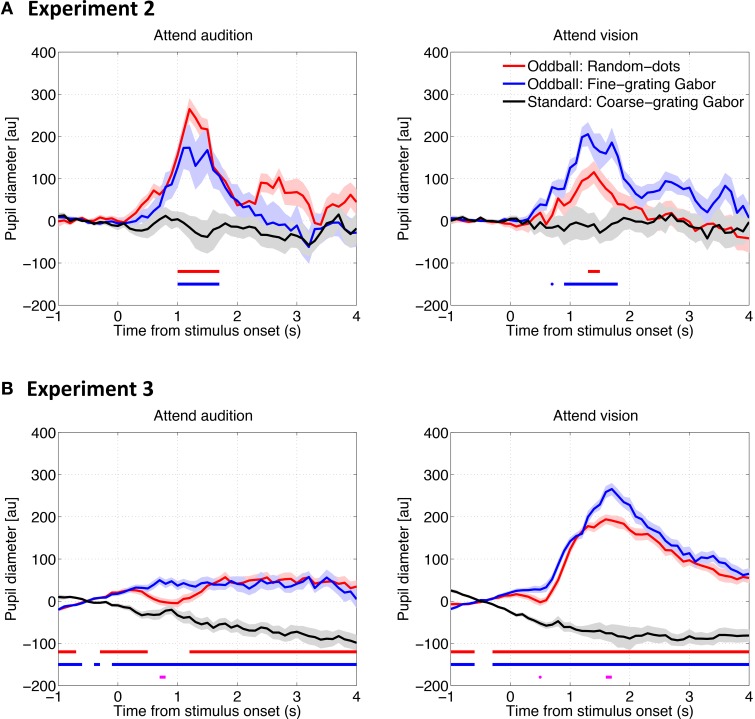
**Pupil diameter change as a function of time relative to the visual stimulus onset in Experiment 2 (A) and Experiment 3 (B)**. The solid lines represent the median of the pupil diameter derived from all trials for all participants as a function of time relative to the visual stimulus onset. The shadows represent the standard error derived from a bootstrapping procedure (resampling *n* = 1500). The horizontal red and blue lines represent statistical differences between the baseline (i.e., the response to the standard coarse-grating Gabor patch) and the Random-dots noise disk and fine-grating Gabor oddballs, respectively. The horizontal magenta line represents the difference between the two types of oddballs (bootstrapping, with the Bonferroni correction).

**Figure 5:**
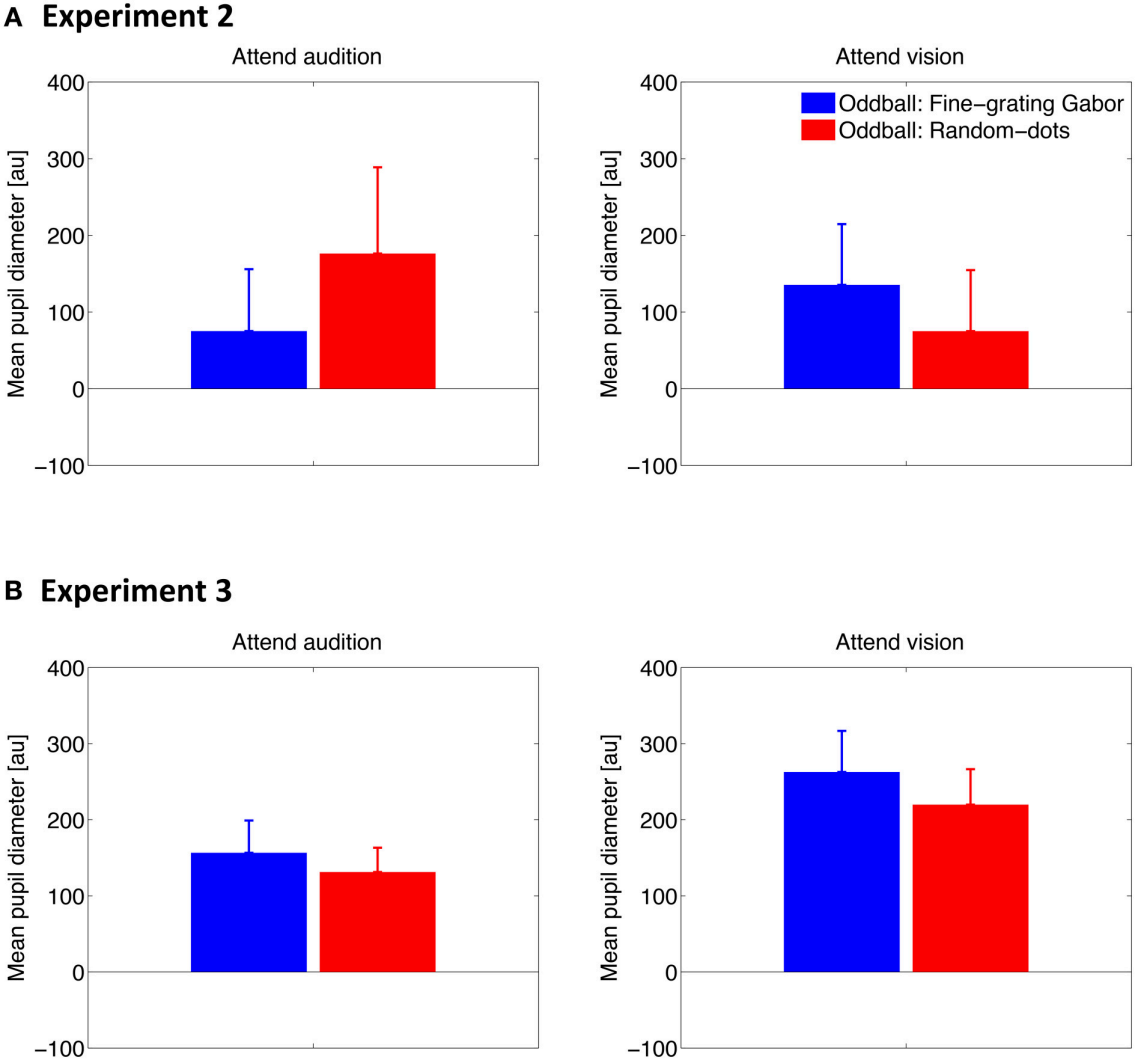
**Mean of the pupil diameter over 0–4 s after the stimulus onset across participants in Experiment 2 (A) and Experiment 3 (B)**. Error bars represent standard errors across participants.

To examine how pupil responds to the deviant visual stimuli and whether attention plays a role there, we conducted the same analyses as describe above except for that the pupil diameter data was time locked to the visual stimulus onset. The time series results are shown in Figure 4. Similar to the pupillary response to auditory stimuli, PDR was found for both types of visual oddballs, when a task was required (i.e., Experiments 2 and 3). However, whether and how the PDR for visual oddballs differed between the oddball types depended on the attention condition, as well as whether or not the visual oddballs were presented synchronously with the auditory oddballs. As shown in Figure 5A, when the visual oddballs were presented synchronously with the auditory oddballs, PDR was found stronger for the fine-grating Gabor patch than random-dots noise disk when attending the visual oddballs, whereas a reverse pattern of result was found when attending the auditory oddballs [*F*_(1, 7)_ = 5.92, *p* < 0.05]. By contrast, when the visual oddballs were presented asynchronously with the auditory oddballs (Figure 5B), no such interaction was found [*F*_(1, 17)_ = 0.35, *p* = 0.56]. PDR was stronger for the fine-grating Gabor patch than random-dots noise disk [*F*_(1, 17)_ = 4.56, *p* < 0.05], regardless of the attention condition. In both experiments, the mean PDR did not differ between the attention conditions [*F*_(1, 7)_ = 0.27, *p* = 0.62 in Experiment 2; *F*_(1, 17)_ = 3.85, *p* = 0.07 in Experiment 3].

The overall results suggested that there was no consistent PDR for the two types of visual oddballs, depending on the attention conditions and the way the visual oddballs were presented in relation to the auditory oddballs. In any case, since the content of the visual oddballs were unrelated to that of the auditory oddballs, the results of the consistently stronger PDR for the auditory noise oddballs than tone oddballs cannot be explained by the PDR for the visual oddballs.

### Behavioral results

**Figure 6:**
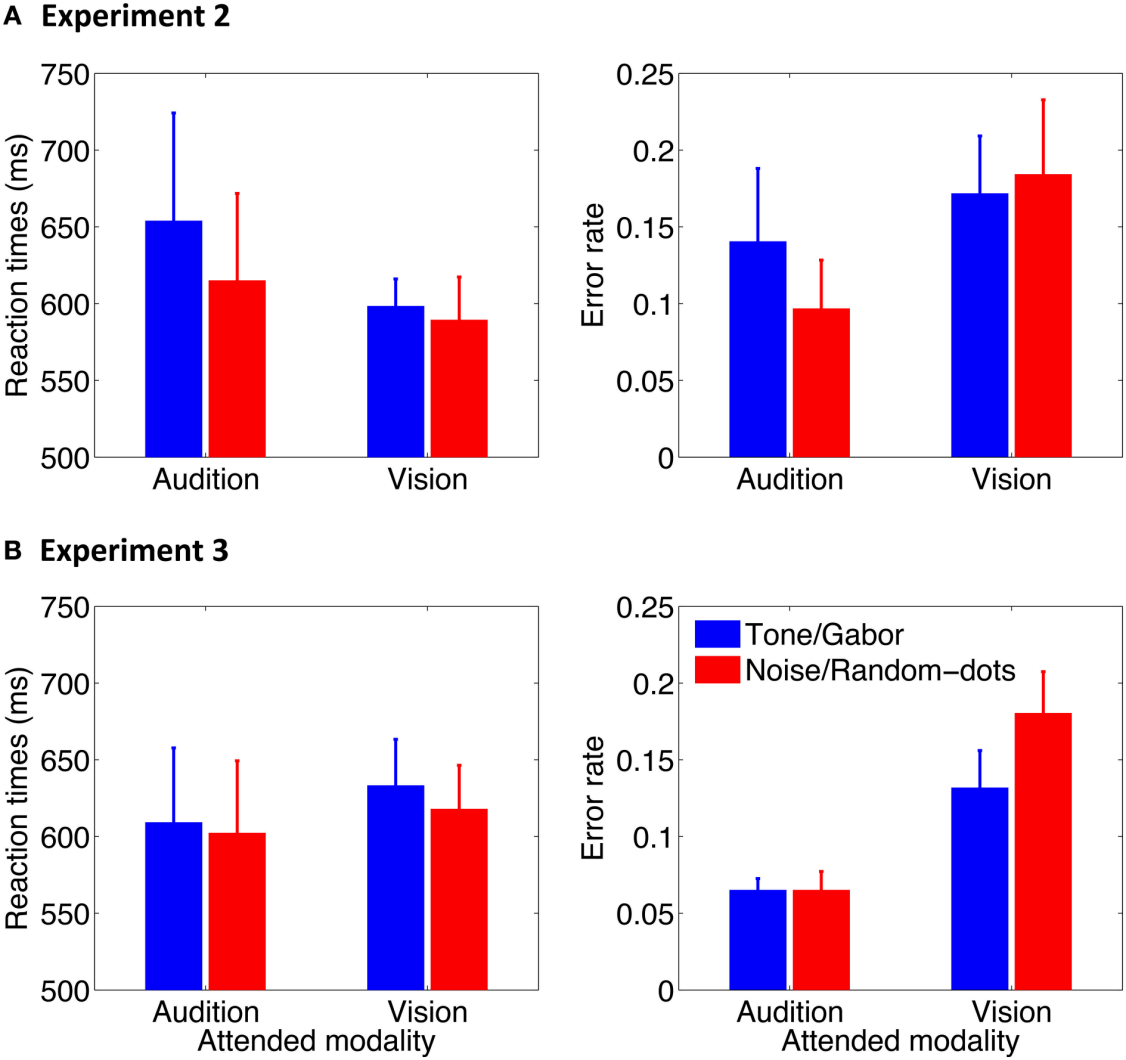
**Mean reaction times and error rate as a function of attention condition in Experiment 2 (A) and Experiment 3 (B)**. Error bars represent standard errors across participants.

Mean correct reactions times and error rates are shown in Figure 6. Data were subjected to a two-way repeated-measures analysis of variance (ANOVA) with oddball type (2000-Hz, noise) and attention condition (attend audition, attend vision) as within-subjects factors. No effect was found in reaction times in Experiment 2 (*F*s < 3.42, *p*s > 0.11) or Experiment 3 (*F*s < 2.44, *p*s > 0.13), whereas the error rates were higher in the attend-vision than in the attend-audition condition in both experiments [*F*_(1, 7)_ = 8.13, *p* = 0.03 in Experiment 2; *F*_(1, 17)_ = 18.47, *p* < 0.001 in Experiment 3]. Neither an effect of the oddball type [*F*_(1, 7)_ = 1.30, *p* = 0.29] nor a two-way interaction [*F*_(1, 7)_ = 0.70, *p* = 0.43] was significant in Experiment 2. In contrast, an effect of the oddball type was significant in Experiment 3 [*F*_(1, 17)_ = 5.28, *p* = 0.03], presumably because of the higher error rates for responding to the random dots than the Gabor in the attend-vision condition [*t*_(17)_ = 2.05, *p* = 0.03], although an interaction just reached marginal significance [*F*_(1, 17)_ = 3.77, *p* = 0.07]. The error rates for the noise and tone were similar to each other.

The overall results indicate that there was no speed-accuracy trade-off. Participants made more errors when performing the visual task than the auditory task. Moreover, they might have made more errors responding to the visual random-dot disk than the fine-grating Gabor when the visual oddballs were presented asynchronously with the auditory oddballs. Most importantly, the behavior responses to the auditory noise and tone oddballs did not differ in reaction times or accuracy. This suggests that the accuracy difference between the attention conditions was not reflected in pupillary responses in which the PDRs were larger for the noise oddballs than for the 2000-Hz tone oddballs, regardless of the attention condition, i.e., task difference (cf. Hyönä et al., 1995).

### Effect of blinks

**Figure 7:**
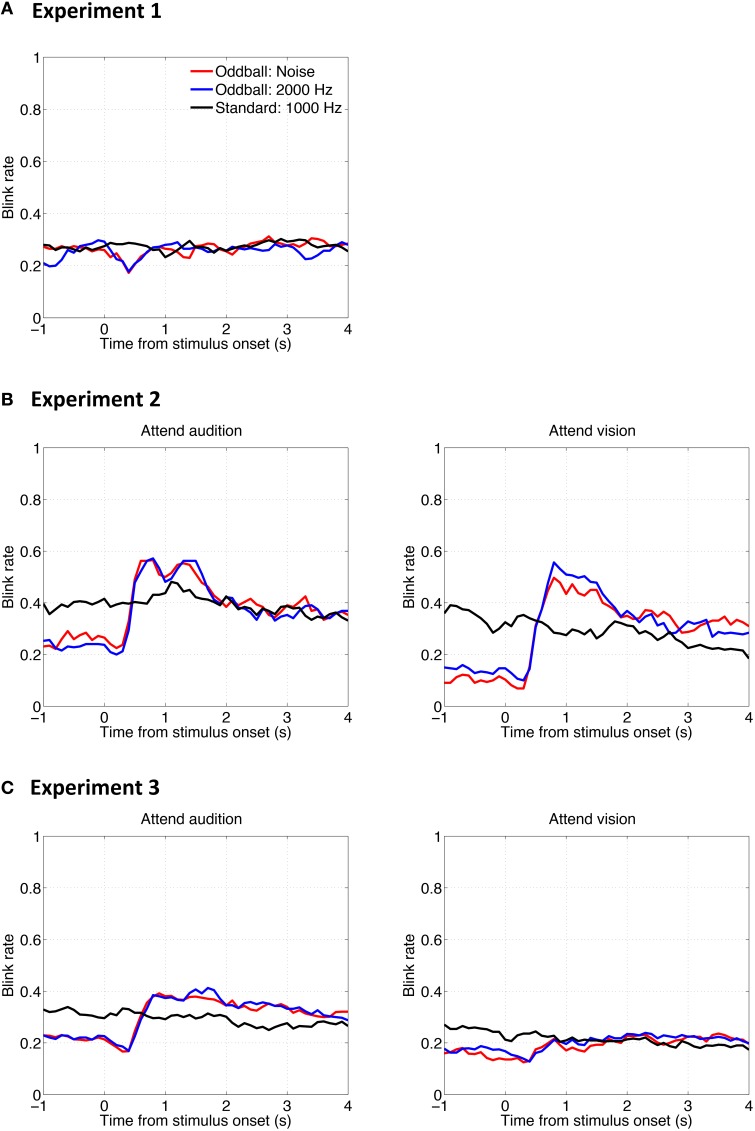
**Mean blink rate (probability of occurrence) as a function of time relative to stimulus onset in Experiment 1 (A), Experiment 2 (B), and Experiment 3 (C)**.

One might suspect that the results presented so far do not reflect the sensitivities of PDRs to auditory stimuli *per se*, but instead are artifacts of eye-blinking, the probability of which might have been modulated by the auditory stimuli. To examine whether auditory oddballs affect blinks and whether blinks affect the PDR, we analyzed the blink occurrence rate as a function of time relative to stimulus onset. Results are shown in Figure 7. The overall pattern of the results shows that the auditory oddballs did affect blinks, but the effect could be either inhibited (Figure 7A) or facilitated (Figures 7B,C), depending on task involvement.

In any case, the PDRs to auditory oddballs cannot be explained by blinks. On one hand, according to the comparison across experiments, larger PDRs to the noise oddball than to the 2000-Hz tone oddball were consistently found, whereas there were inconsistent blink patterns among the three experiments. On the other hand, on comparison within the same experimental setup, the noise oddball and the 2000-Hz tone oddball elicited similar blink results (red lines vs. blue lines in Figure 7), whereas different PDRs were induced (red lines vs. blue lines in Figure 2).

The blink rate functions exhibited other interesting patterns that depended on the experimental parameters, such as involvement in the task at hand. However, elaborating on those patterns is beyond the scope of the current study.

### Gaze positions

**Figure 8:**
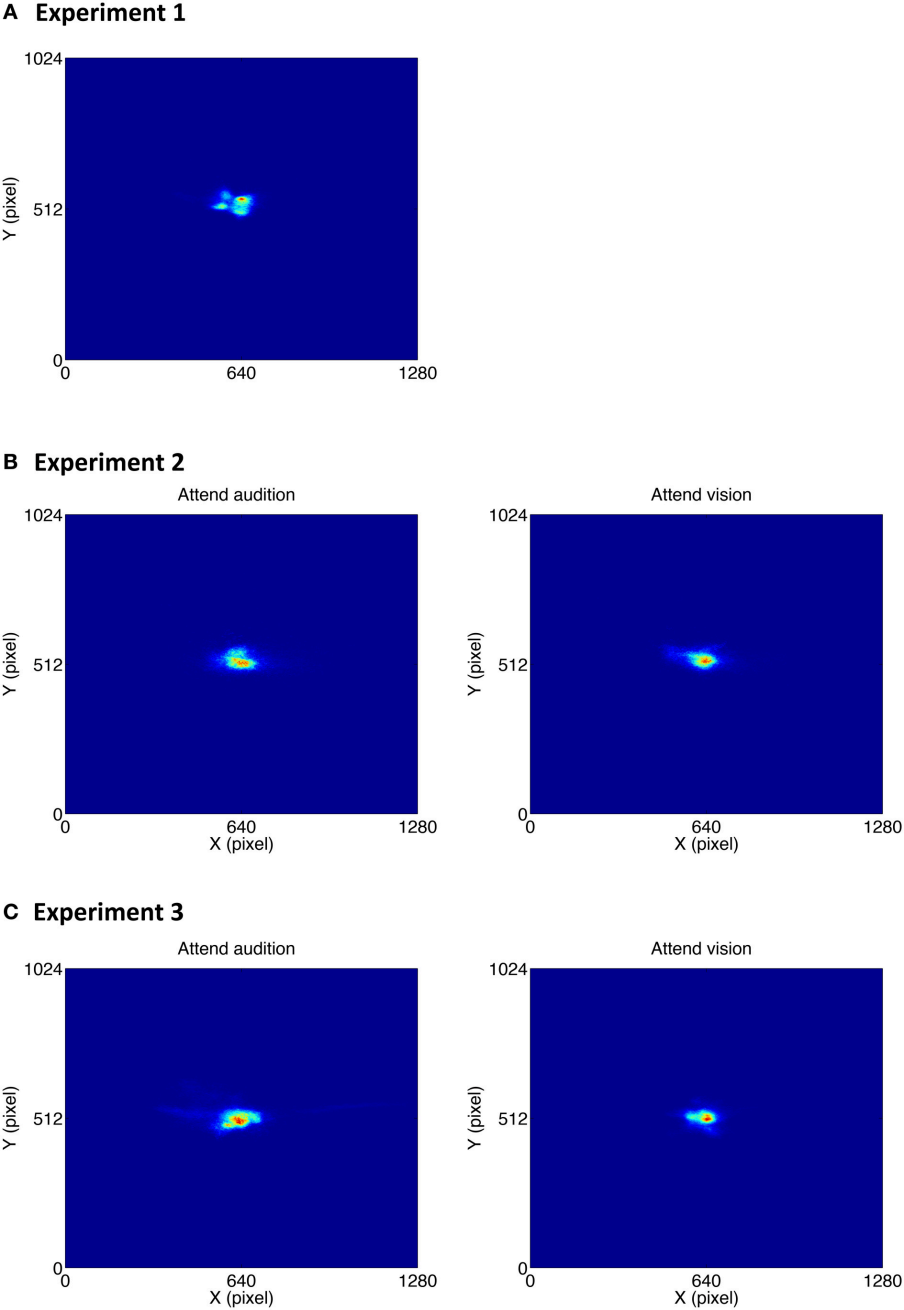
**Intensity map of the gaze positions in Experiment 1 (A), Experiment 2 (B), and Experiment 3 (C)**.

Although participants were asked to fixate at the center of the screen throughout the experiment, it is unclear how exact they were in following the instruction, in particular when it took quite an amount of time to complete the experiment, i.e., around 20 min. Furthermore, the visual stimulus differed among the experiments: in Experiment 1, it was a small fixation point, whereas in Experiments 2 and 3, it was a disk that occupied quite a large space, i.e., 5° by 5° of visual angle. It is important to confirm the gaze position since gaze position could influence the accuracy of the pupil size measurement in a video-based eye tracking system as used in the current study. We therefore analyzed gaze position in which the gaze positions throughout the three experiments were summed up across all participants. The intensity maps of the gaze position are shown in Figure 8.

To examine whether the deviation of the gaze position from the center point differed across experiments, the mean distance between the gaze position and the center point was calculated for each participant, and all the data were subjected to a between-subjects ANOVA. Results showed that the gaze deviation distance did not differ among the experiments [mean distances were 104, 152, and 112 pixels in Experiment 1 to 3, respectively; *F*_(2, 33)_ = 0.64, *p* = 0.53].

To examine whether the attention condition influenced the gaze pattern, the mean gaze deviation distance in Experiments 2 and 3 was subjected to a mixed ANOVA with attention condition (attend audition, attend vision) as within-subjects factor and oddball synchrony (synchronous, asynchronous) as between-subjects factor. Results showed that the gaze deviation distance was smaller in the attend vision condition than in the attend audition condition [mean distances were 90 and 160 pixels, respectively; *F*_(1, 24)_ = 8.29, *p* = 0.008]. The effect did not interact with the oddball synchrony condition [*F*_(1, 24)_ = 0.25, *p* = 0.62]. The overall results suggest that gaze position was more accurately focused at the center when participants performed the visual task, i.e., the attend vision condition, than when they performed the auditory task, regardless of whether the visual and auditory oddballs were presented synchronously or not.

Even though there was difference in the gaze position between the attend audition and attend vision condition, approximately 1.4°, the PDRs to the auditory oddballs were consistently found in these two conditions. Furthermore, the gaze positions did not differ among the three experiments, indicating that participants did follow the instruction to focus on the center point, regardless of the stimulus type presented at the center. The pupillary responses were measured consistently with the gaze position controlled.

## Discussion

In the three experiments, we showed PDRs to deviant auditory noise bursts against a background of repetitive pure tone presentation. Moreover, the PDRs to the deviant auditory events were stronger for the noise bursts than the tones oddballs. The overall results indicate that PDR is not only sensitive to the acoustic change, but also the content of the change. They suggest that the human PDR is used as a physiological index of the orienting reflex to the detection of a novel and salient auditory event. The current study extends our understanding of the PDR to auditory salience, defined by novelty and uniqueness, deviating from the background.

The effect of PDRs to the novel and salient auditory event remains robust regardless of whether top-down attention is focused on or away from the auditory stimuli. The results are in line with the evidence that is obtained from an EEG study in which the early novelty P3 is insensitive to attentional manipulations (Escera et al., 1998), and suggest that the underlying mechanism is the change-detector mechanism that is related to stimulus-driven attention orienting. It is also known from attention research that a noise burst attracts attention and can affect visual task performance even when attention is top-down focused on a visual task (Koelewijn et al., 2009). Together with our findings, the overall results suggest that a noise burst is a salient event, which attracts attention when it deviates from the background. Most importantly, the effect of attentional capture by the deviant and salient noise burst is reflected in the PDR.

Why does pupil respond to the noise oddballs differently from the tone oddballs? Noise oddballs elicited stronger PDRs than tone oddballs against a background of the repetitive presentation of tones at lower frequency. The results may be taken as indicating an asymmetry in terms of the difference in the spectral content of the stimuli. The following explanations may reflect mechanisms at different levels of auditory processing and are not mutually exclusive. One explanation is that in the noise oddball conditions, the noise oddball activates a wide range of frequency channels at a certain level of auditory processing, in addition to a few channels activated already by the standard tones. In the tone oddball conditions, the tone oddball activates a small number of channels, which do not differ in number from ones activated by the standard tones of lower frequency. It may be that the PDR increases with the number of newly activated channels. Another explanation is based on a property of the superior colliculus: Animal physiological studies suggest an involvement of the superior colliculus in the pupillary responses (Netser et al., 2010; Wang et al., 2012). The noise preference of the PDR may reflect activities of auditory neurons in the superior colliculus, which are known to respond more robustly to broadband than to narrowband stimuli (Wise and Irvine, 1983; King and Carlile, 1994). We can offer another explanation in terms of stimulus loudness, and/or salience. Although the stimulus intensity of the noise and tone was the same in A-weighted sound pressure level (65 dB), the noise was still louder than the pure tones (80.7 phons for the white noise, 57.7 phons for the 2000-Hz pure tone, and 62.4 phons for the 1000-Hz pure tone, estimated according to Glasberg and Moore, 2006). Indeed, it is shown that PDR reflects loudness, as well as subjective salience of sounds in more of a psychological sense (Liao et al., 2015).

One may notice that, overall, the PDRs in Experiments 2 and 3 (normalized pupil diameter of ~200 au) were stronger than in Experiment 1 (~100 au). This could be because the demands of the task enhanced general cognitive processes on the stimuli (Hyönä et al., 1995; Koelewijn et al., 2015) or due to the involvement of decision making (Einhäuser et al., 2010). The observed PDRs to auditory oddballs in Experiments 2 and 3 may thus be confounded with other cognitive processes rather than their being just a purely stimulus-driven auditory stimulation. Although there was the possibility of the involvement of other cognitive processes in Experiments 2 and 3, the finding of larger PDRs to the noise oddballs than to the tone oddballs suggests that PDRs to auditory oddballs are sensitive to the stimulus properties, regardless of task involvement.

Apparently, there was not a marked attentional modulation of the PDR to auditory oddballs. This is backed up by the results of the two experiments in which the auditory oddballs were presented synchronously and asynchronously with the visual oddballs. In any case, the content of the auditory oddballs was unrelated to the visual oddballs, and thus should have been ignored when the task was to discriminate the visual oddballs. Under this circumstance, the PDR was still stronger for the noise than for the tone oddballs, the same as when attending to the auditory oddballs *per se*. The overall results suggest that the PDR could be a physiological marker for the orienting reflex to a deviant auditory stimulus, which is independent of attention to sensory modalities. A caveat is that this does not imply that top-down attentional control is unable to modulate the PDR to a deviant auditory stimulus. The finding that PDRs were in general larger when a task was involved suggests that attention and/or cognitive effort modulates PDRs. It is also well understood that a top-down control setting can modulate involuntary orienting to visual stimuli in behavior (for a review, see Theeuwes, 2010) and eye movement measurements (for a review, see van der Stigchel et al., 2006). Further research is required in order to determine whether and how top-down attentional control plays a role in the PDR to auditory stimulation.

The current finding of PDRs to auditory oddballs should be added to the short list of physiological responses to rare auditory events such as mismatch negativity, novelty P3 response, and neural responses related to stimulus-specific adaptation. The underlying mechanism among these measurements may be different but yet related. The relationship between mismatch negativity and the N-methyl-D-aspartate (MNDA) receptors (Javitt et al., 1996; Kreitschmann-Andermahr et al., 2001; Heekeren et al., 2008) is more fully established, but it still remains unclear in other neuropharmacological systems (see review by Garrido et al., 2009). Since pupillary responses are known to reflect modulation of the LC-NE system (Aston-Jones and Cohen, 2005), the results that the characteristics of PDRs to the acoustic novelty and change are similar to those of the novelty P3 responses suggest that the neurotransmitter associating with the novelty P3 response is related to norepinephrine pathways (cf. Polich, 2007). The neural substrate of stimulus-specific adaptation along the auditory pathway is found in the auditory cortex (Javitt et al., 1994; Ulanovsky et al., 2003) and inferior colliculus (Patel et al., 2012), which may share a common underlying mechanism of the PDRs to auditory stimulation.

In summary, pupil dilation can be used as a physiological marker for detection of deviant auditory stimuli. The interactions among stimulus properties and task involvement are critical in determining the PDR to a deviant auditory stimulus. Attention to sensory modalities appears not to be a critical factor for the stimulus-dependent PDR.

## Author contributions

HL, MK, and SF developed the study concept. All authors contributed to the study design. HL, MY, and SK conducted experiments and collected data. HL analyzed data and drafted the manuscript. SF provided critical revisions. MY, SK, and MK provided critical comments. All authors approved the final version of the manuscript for submission.

### Conflict of interest statement

The authors declare that the research was conducted in the absence of any commercial or financial relationships that could be construed as a potential conflict of interest.
